# Careful patient selection together with optimal implant positioning may reduce but does not eliminate the risk of elevated serum cobalt and chrome levels following metal-on-metal hip resurfacing

**DOI:** 10.1177/11207000221124302

**Published:** 2022-10-30

**Authors:** Alexander Oxblom, Håkan Hedlund, Raed Itayem, Li Felländer-Tsai, Mathias Vidgren, Ola Rolfson, Harald Brismar

**Affiliations:** 1 Division of Orthopaedics and Biotechnology, CLINTEC, Karolinska Institute, Stockholm, Sweden; 2 Department of Orthopaedics, VO KOU, Sodertalje Hospital, Sodertalje, Sweden; 3 Department of Orthopaedics, Visby Hospital, Visby, Sweden; 4 Department of Orthopaedics, Institute of Clinical Sciences, Sahlgrenska Academy University of Gothenburg, Sweden; 5 Department of Reconstructive Orthopaedics, Karolinska University Hospital, Stockholm, Sweden

**Keywords:** Hip arthroplasty, hip resurfacing, metal ions, metal-on-metal

## Abstract

**Background:**

Elevated serum chrome (sCr) and cobalt (sCo) concentrations are associated with local tissue adverse reactions to metal debris following metal-on-metal hip resurfacing (MoM-HR). Serum metal ions <2 µg/l are probably of little clinical relevance and a pragmatic “safe” threshold <5 µg/l has been suggested.

The primary aim of this study was to evaluate if a careful selection of patients combined with optimal implant positioning could eliminate cases with “unsafe” serum metal ion levels. A secondary aim was to study the association between different risk factors and having Co and/or Cr levels >5 µg/l.

**Patients and methods:**

This is a retrospective, single-institution cohort study of 410 consecutive patients operated on with a Birmingham Hip Resurfacing (BHR) implant between 2001 and 2014. 288 of these had a unilateral MoM-HR, pelvic and true lateral radiographs, and a related sCo and sCr sample, and were included in the final analysis. They were allocated to either a presumed “optimal group” consisting of only men aged <60 years old, with femoral head component >48 mm diameter, and with a cup positioned within Lewinnek’s safe zones, or a “suboptimal group” consisting of the remaining patients. Fisher′s exact test and multiple logistic regression analyses were performed.

**Results:**

In the optimal group 48% (47/97) had serum metal ions >2 µg/l and 8% (8/97) >5 µg/l compared to 61% (116/191) and 18% (34/191) in the suboptimal group, *p* = 0.059 and *p* = 0.034 respectively. Acetabular cups with an anteversion <5 degrees had the highest odds ratio, 6.5 (95% CI, 3.0–14.3), of having sCo and sCr concentrations exceeding 5 µg/l.

**Conclusions:**

A well oriented BHR acetabular component in a presumably “optimal” patient reduces the risk of having elevated serum metal ions but does not eliminate it. Insufficient cup anteversion seems to be the strongest associated factor of elevated serum metals.

## Introduction

When introducing metal-on-metal hip resurfacing (MoM-HR), the potential advantages compared to conventional total hip arthroplasty (THA) were a reduced risk of dislocation due to a big-sized caput,^1^ a bone-preserving technique resulting in less altered biomechanics of the joint,^2^ and therefore a better functional outcome.^3^ Another important rationale for MoM-HR was to eliminate the risk of implant loosening associated with polyethylene wear in young and active patients receiving THA.^4^ However, even if minute, the cobalt (Co) and chrome (Cr) wear of MoM-HR resulted in local tissue adverse reactions to metal debris (ARMD) in some patients.^5,6^

ARMD is a collective term including tissue metallosis, aseptic lymphocytic vasculitis associated lesions (ALVAL), and pseudotumours – solid or cystic periprosthetic tissue masses.^7^

The risk of developing pseudotumours appears to be higher in patients with high serum cobalt (sCo) and serum chrome (sCr) concentrations although a low serum concentration does not eliminate the risk of pseudotumour formation.^8^ Blood metal concentrations of more than 5 µg/l have been suggested, although it is still debatable, as a threshold of increased risk of ARMD;^9^ others have suggested even lower values.^10^ Of note is that any foreign metal object placed in the body is likely to release metal ions and that serum metal ion concentration is merely a reflection of the intra-articular level which can be up to 100 times that in serum,^5^ a relationship that varies across individuals.

To reduce the risk of revision following MoM-HR, caution has been recommended in females and patients with femoral heads <50 mm.^11,12^ However, it has been suggested that female sex per se is not a contra-indication considering that women as a group have smaller femoral heads than males.^13^ MoM-HR has not been advised for the elderly, since bone quality is believed to be crucial using the technique,^14^ and the survivorship of a conventional THA in this patient group is excellent.^15^ In addition, the positioning of the acetabular cup seems to be critical.^16^ An abduction angle >55° and an anteversion angle <10° have been associated with increased metal wear and subsequently increased systemic Co and Cr levels.^17–19^

The primary aim of this study was to evaluate if a careful selection of patients combined with optimal implant positioning can eliminate the risk of “unsafe” serum metal ion levels. A secondary aim was to study the association between different risk factors and having Co and/or Cr levels >5 µg/l.

## Patients and methods

This is a retrospective single-institution cohort study of all 410 consecutive patients operated on with a Birmingham Hip Resurfacing (BHR) (Smith & Nephew, Andover, Massachusetts, USA) at Karolinska University Hospital, between 2001 and 2014 by 2 experienced hip arthroplasty surgeons using a posterior approach. The follow-up period was November 2001 to December 2019. All data were retrieved from electronic patient records (CGMtakecare, CompuGroup Medical). The study was approved by the Regional Ethical Review Board in Stockholm (2017/1841-31/2).

**Figure 1.**
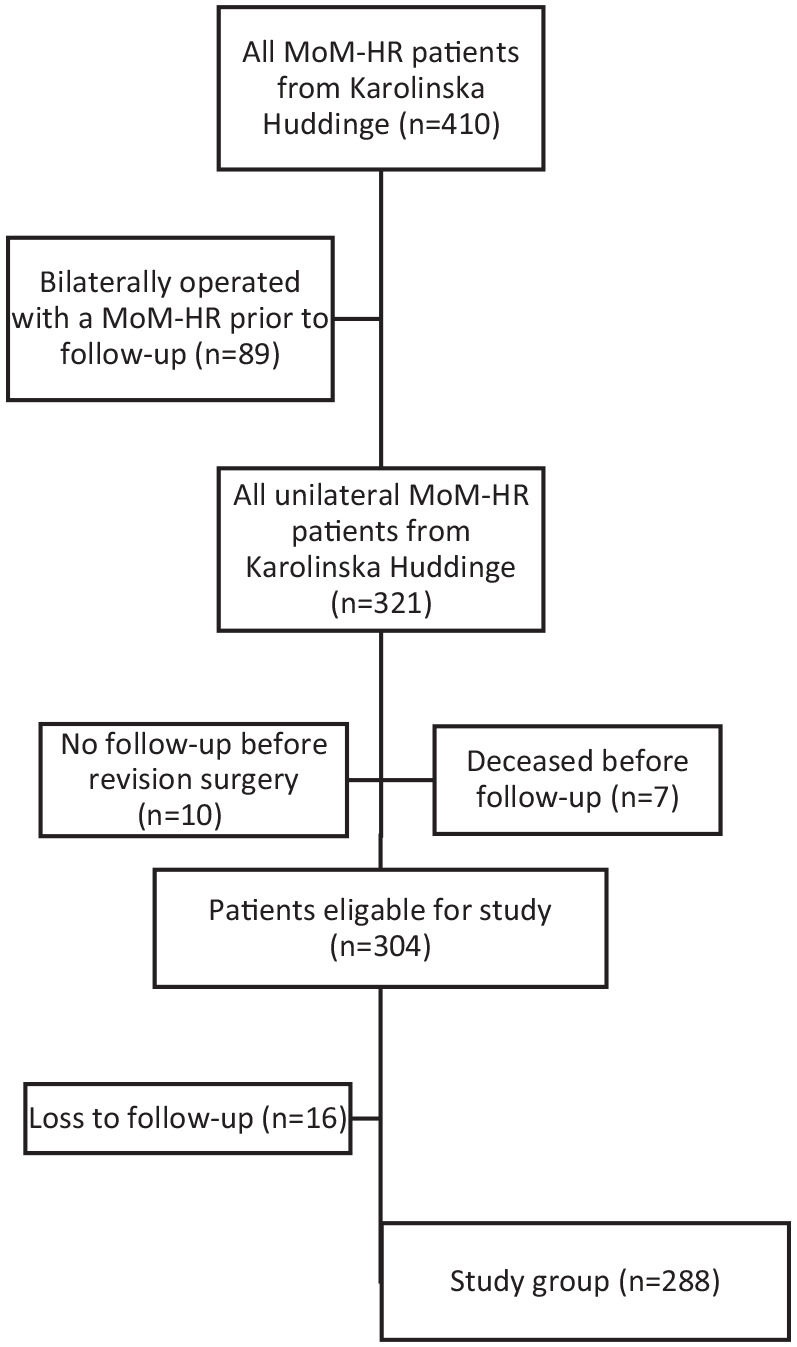
Flowchart of patient selection.

The inclusion criteria were: the presence of a unilateral operated MoM-HR, in this study a BHR; and 1 complete follow-up defined as having an anteroposterior (AP) pelvic view and a cross-table lateral view; and sCo and sCr concentrations. At the study start in 2019, the patients’ electronic medical records were analysed together with present x-rays. Patients who had been operated on before follow-up with a contralateral MoM-HR (*n* = 89), were deceased (*n* = 7), or had undergone revision (*n* = 10) or who had never been radiologically examined or had their serum metal ions taken were excluded. Of the remaining 304 patients, follow-up was incomplete in 16 patients, leaving 288 patients in the final study group (Figure 1).

The standard follow-up routine for MoM-HR patients at our institution consisted of both a hip x-ray and metal ion analysis at 1 year, 5 years, and 10 years postoperatively. Those with either abnormal findings on x-rays or elevated metal ion concentrations (sCo and/or sCr >5 µg/l) were offered more frequent investigations.

Using the “Ortho Toolbox”-instrument on a SECTRA workstation (PACS, Sectra AB, Linköping, Sweden) 2 radiographic measurements were performed: cup inclination angle and cup anteversion angle. All radiographic measurements were carried out by the first author (AO), a 4th-year orthopaedic resident. Measurements were performed blinded, i.e. the assessor did not know individual serum cobalt or chrome levels, nor was he involved in the surgeries or in the follow-up of patients.

The inclination angle was calculated by measuring the angle between a line drawn between the ischial tuberosities and a line drawn between the 2 most distant edges of the AP elliptic projection of the cup. The version of the cup was defined as the angle composed by a line between the most distant edges of the lateral elliptic projection of the cup and a line drawn perpendicularly to the table on the cross table view.^20^ Both the anteversion angle and the inclination angle were categorised according to Lewinnek’s safe zones into; optimal cup positioning i.e. 5–25° of anteversion and 30–50° of inclination, and sub-optimal implant positioning, i.e. those outside the optimal ranges.^21^ In the case of repeated x-ray imaging and serum metal analysis, the last hip x-ray performed with a concomitant serum metal ion analysis was used.

Blood samples of Co and Cr were collected with the first vial discarded to avoid metal contamination from the needle. After coagulation at room temperature, the blood was centrifuged to separate the serum. The samples were analysed at ALS Scandinavia, Lulea, Sweden, with mass spectrometry using ICP-SFMS technology. 2 thresholds for “safe” metal ion concentrations were used, 2 µg/l and 5 µg/l.

Patient factors investigated were sex, age, and femoral resurfacing head component size. Finally, based on previous research, we defined an “optimal group” consisting of only men,^22,23^ younger than 60 years,^24^ with femoral head components >48 mm in diameter,^17^ with optimal cup positioning,^25^ and a “suboptimal group” consisting of the remaining patients.

### Statistical analysis

To ensure the inter-rater reliability of our radiological measurements we used a random number generator to randomise 50 patients in the cohort for the intraclass correlation (ICC) analysis. Radiological angle measurements of cup anteversion and cup inclination were performed independently by the first (AO) and last (HB) authors on the randomised 50 patients. The analysis of ICC was 2-way random, single measure, absolute agreement.

The odds ratio (OR) for having Co and/or Cr levels >5 µg/l was analysed separately for each variable using simple logistic regression followed by multiple logistic regression to create a model adjusted for the effect of the different variables. *P*-values of numeric variables were calculated either by independent *t*-test (symmetrically distributed variables) or by Mann-Whitney U-test (skewed variables). Comparisons between categorical variables were analysed using Fisher’s exact test. A *p*-value <0.05 was considered statistically significant. R version 3.6.3. and STATA 16 were used for statistical analyses.^26,27^

## Results

The mean age at surgery was 51 years (range 27–71 years) and 68 of 288 (24%) patients were women. Men had a bigger median femoral head component size than women (52 vs. 46 mm, *p* < 0.001). Women had higher median sCo and sCr concentrations than men (Table 1). Patients with femoral head components <50 mm had higher median sCo (1.52 µg/l, interquartile range [IQR] 1.05–2.51, vs. 1.17 µg/l, IQR 0.82–1.57, *p* = 0.002) and sCr levels (2.65 µg/l, IQR 1.72–4.13, vs. 2.20 µg/l, IQR 1.40–3.11, *p* = 0.005) compared to those with femoral head components ⩾50 mm. Cup positioning did not seem to differ between men and women. 53% of all patients had their acetabular cups positioned within Lewinnek’s safe zone.

**Table 1.** Patient demographic characteristics.

Characteristic	Cohort	All patients	Males	Females	*p*-Value
Number of patients		288	220	68	NA
Mean age, years (range)		51 (27–71)	52 (27–71)	50 (28–69)	0.26^a^
Mean follow-up time, years (range)		7.8 (1–16)	7.6 (1–16)	8.4 (2–15)	0.14^a^
Median femoral head component size, mm (IQR)		52 (48–54)	52 (50–54)	46 (46–48)	<0.001^b^
Median serum cobalt concentration, µg/l (IQR)		1.25 (0.85–1.82)	1.14 (0.81–1.57)	1.58 (1.20–2.44)	<0.001^b^
Median serum chrome concentration, µg/l (IQR)		2.20 (1.45–3.56)	2.02 (1.41–3.10)	2.80 (1.91–4.10)	0.001^b^
Mean inclination angle, degrees (range)		42 (22–60)	42 (22–60)	43 (29–59)	0.13^a^
Inclination angle in degrees, cohort (%)	30–50°	236 (82)	184 (84)	52 (76)	0.084^c^
	<30°	15 (5)	13 (6)	2 (3)	
	>50°	37 (13)	23 (11)	14 (21)	
Mean anteversion angle, degrees (range)		15 (−18–49)	15 (−18–49)	17 (−17–38)	0.32^a^
Anteversion angle in degrees, cohort (%)	5–25°	180 (63)	139 (63)	41 (60)	0.66^c^
	<5°	47 (16)	37 (17)	10 (15)	
	>25°	61 (21)	44 (20)	17 (25)	

IQR, interquartile range; NA, not applicable.

a Independent *t*-test.

b Mann-Whitney U-test.

c Fisher’s exact test.

**Figure 2.**
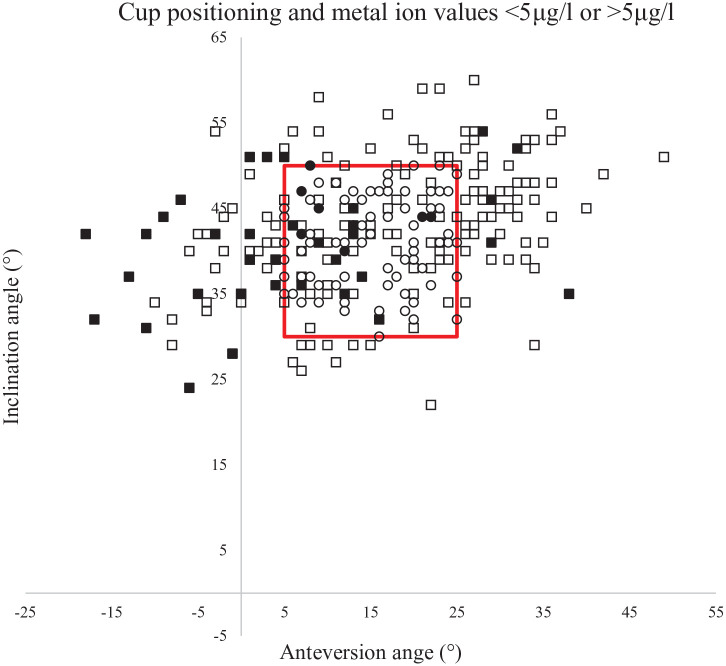
Scatterplot showing the cup orientation of the patients in the study. Circular marks represent individual patients in the “optimal” group and squares represent individual patients in the “suboptimal” group. Filled marks represent patients with metal ions >5 μg/l and unfilled those with <5 μg/l. The red box marks values within Lewinnek safe zone (anteversion angle 5–25°, inclination angle 30–50°). Note the high proportion of filled squares when the anteversion angle is less than 5 degrees.

There were 97 patients in the “optimal” group (all men, mean age 49 years, range 33–59 years, median femoral head component size 52 mm) and 191 in the “suboptimal” (123 men, mean age 52 years, range 27–71 years, median femoral head component size 50 mm). 8% of the patients with presumably optimal characteristics and cup positioning (according to Lewinnek’s safe zones) had concentrations of 1 or both metal ions >5 µg/l and 48% >2 µg/l. In the “suboptimal group” 18% had concentrations >5 µg/l and 61% more than 2 µg/l (Table 2) (Figure 2).

**Table 2.** “Optimal group” (Men that <60 years old, with a femoral head size >48 mm, and an ideal cup positioning according to Lewinnek safe zones) versus “Suboptimal group” (Lacks 1 or more of the criteria’s necessary to be included in the “Optimal group”).

Characteristic	Optimal group	Suboptimal group	*p*-Value
Number of patients (%)	97 (34)	191 (66)	NA
Mean follow-up time, years (range)	7.6 (2–16)	7.9 (1–16)	0.46^‡^
Median cobalt, µg/l (IQR)	1.07 (0.78–1.43)	1.35 (0.93–2.0)	0.10^†^
Median chrome, µg/l (IQR)	1.94 (1.39–2.66)	2.37 (1.54–3.90)	0.22^†^
Number of patients with serum cobalt and/or chrome over 2 µg/l (%)	47 (48)	116 (61)	0.059^¥^
Number of patients with serum cobalt and/or chrome over 5 µg/l (%)	8 (8)	34 (18)	0.034^¥^

IQR, interquartile range; NA, not applicable.

† Mann-Whitney U-test, ^‡^Independent t-test, ^¥^Fisher’s exact test.

Cup anteversion <5° was associated with an increased risk of sCo and/or sCr >5 µg/l (OR 6.5; 95% CI, 3.0–14.3) when compared to the ideal cup position (Table 3). Female sex and older age were also to some degree associated with increased risk of sCo and/or sCr >5 µg/l (OR 2.2; 95% CI, 1.0–4.8, and 1.1; 95% CI, 1.0–1.1 respectively).

**Table 3.** Simple and multiple logistic regression analysis of the odds ratio (OR) of having serum cobalt and/or chrome concentration >5 µg/l.

Characteristic	Cohort	Crude OR (CI)	*p*-Value (crude)	Adjusted OR (CI)	*p*-Value (adjusted)
Age		1.04 (1.00–1.08)	0.061	1.05 (1.00–1.09)	0.034
Sex (woman)		1.78 (0.86–3.57)	0.11	2.22 (1.00–4.83)	0.045
Femoral head component size		0.98 (0.90–1.08)	0.71	NA	NA
Year of surgery		0.90 (0.80–1.00)	0.048	NA	NA
Follow-up time		1.09 (0.99–1.20)	0.088	NA	NA
Inclination angle	<30°	1.49 (0.33–4.97)	0.56	1.50 (0.30–5.88)	0.58
	>50°	0.93 (0.30–2.37)	0.89	1.21 (0.36–3.46)	0.74
Anteversion angle	<5°	6.11 (2.87–13.9)	0.010	6.45 (2.96–14.32)	0.010
	>25°	0.80 (0.26–2.12)	0.68	0.70 (0.22–1.93)	0.52

CI, 95% confidence interval; OR, odds ratio; NA, not applicable.

The adjusted OR are adjusted for the other variables used in the multiple logistic regression analysis (age, sex, inclination angle <30°, inclination angle >50°, anteversion angle <5°, and anteversion angle >25°).

The ICCs for inter-rater reliability of measurements on anteversion angle and inclination angle were 0.98 (95% CI, 0.96–0.99) and 0.98 (95% CI, 0.96–0.98) and both were statistically significant (*p* < 0.001).

## Discussion

Careful patient selection combined with optimal implant positioning reduces but does not eliminate the risk of having “unsafe” sCo and/or sCr concentrations following BHR at mean 8 years follow-up. Malpositioning, i.e. insufficient anteversion, seems to be the strongest associated factor of elevated serum metals.

In our cohort, 15 % of all patients had sCo and/or sCr concentrations >5 µg/l. High local Co or Cr concentrations can be toxic, affecting the proliferation and function of osteoblasts and osteocytes,^28^ and may also lead to cell death by apoptosis.^29^ Osteolysis around the implant is a common finding in revised MoM-HR.^30^ High serum metal ion levels are more common in patients with ARMD and among those revised, although there does not seem to be a direct linear relationship between metal ions and ARMD.^8,31^ Metal concentrations in serum <2 µg/l are less common in patients with pseudotumours while concentrations >5 µg/l are frequently found.^9,32^ The individual risk for having a complication related to these “unsafe” values is, however, not known. Hypothetically there may be individual variances in sensitivity to metal ions, and the relation between measured serum values and actual concentration locally is also likely to differ across individuals.

Theoretically, if we had only operated on men <60 years, with femoral head components sized >48 mm, and managed to position their implants in an “ideal” position according to Lewinnek′s safe zones, the proportion of patients with supposed “unsafe” values would have been reduced to 8%. In our study, 48% of the supposedly “optimal” patients had values >2 µg/l which is of some clinical concern. Since even a supposed “optimal” patient with an “ideally” implanted prosthesis may get “possibly unsafe” serum metal ion concentrations, a substantial proportion of these patients will need costly and time-consuming follow-up that is not needed in patients with conventional prostheses.

In our study cohort, an acetabular cup anteversion <5° had the highest association with metal ion concentrations >5 µg/l. This is in accordance with a previously published study reporting that patients with insufficient cup anteversion and high cup inclination had high blood metal concentrations.^18^ Other studies have found a high wear rate in implants subjected to edge loading.^33^ Edge loading occurs when the loaded surface of the femoral metal sphere comes into contact with the edge of the acetabular component. This happens earlier in the range of motion if the implant is malpositioned and will depend on the total range of motion that the individual patient can perform. The theory of the importance of edge loading is further strengthened by the results from a multi-centre and multi-surgeon study on retrieved implants from revised patients, in which edge loading was prominent on implants with >59° inclination.^34^ We did not find any association between excessive anteversion or inclination and elevated metal ions in our cohort. This could be due to the limited number of patients with excessive anteversion or inclination in our study. Another reason could be differences in study design. One could speculate that insufficient anteversion is especially harmful considering that loaded flexion of the hip is a common activity, i.e. in getting up or climbing stairs.

Disappointingly, only 53% of the operated patients in our cohort had their cups positioned within Lewinnek′s safe zone. This result does, however, correspond to previously reported results.^19,33^

We recognise that our study has several limitations. Even though angle measurements on x-rays, in general, are relatively straightforward, both observer error and x-ray projection differences across patients may reduce accuracy and potentially increase the type 2 errors.^35^ The anteversion angle especially, an angle affected by pelvic tilt, is difficult to measure in a standardised manner, since it may be affected by patient positioning and spine deformities such as scoliosis. However, previous studies have shown that the method used in this study is consistent, reliable, and reproducible.^36^ Our ICC results also suggest good reliability in our radiological measurements.

The contact patch to rim distance (CPRD)^37^ has been suggested to be an important radiological parameter in determining the risk of excessive MoM-HR prosthesis wear.^38^ CPRD is the distance between the area where the femur has contact with the acetabular component and the acetabular rim. It is affected by coverage, size, orientation of the acetabular component, and is probably most precisely measured with computed tomography (CT).^39^ In contrast, the use of Lewinnek’s safe zones to determine patients at risk of excessive prosthesis wear could be debated as these zones were primarily developed as a tool for orthopaedic surgeons to minimise the risk of hip prosthesis dislocations.^21^ In addition, the risk of edge-loading in flexion is affected by the combination of the patient’s natural femoral neck anteversion and acetabular anteversion. The mechanical approach using Lewinnek’s safe zones does not take this into account. One could argue that using the transverse acetabular ligament (TAL) for the orientation of the cup in anteversion would be more beneficial.^40^ However, since there is a variation in TAL in relation to the acetabular anteversion, we believe, based on the findings in our study, that it is preferable to position the cup at >5° anteversion, even if the orientation of TAL would suggest a less anteverted cup. The use of the CPRD for assessing risk of excessive wear is probably more accurate than cup anteversion and retroversion angles, but we believe it is less convenient in the clinical situation since radiological follow-up is most common by plain x-ray rather than CT scans.

Age was associated with a higher odds ratio of having serum cobalt and/or chrome concentration >5 µg/l. Reduced kidney function, more often found in older patients, has previously been associated with higher sCo levels,^41^ but more recent studies contradict this.^42^ Another explanation could be a less stable joint in the elderly due to a reduced muscle volume and thereby increased risk of micro separation during the swing phase.^43^ There are several other factors that may also potentially affect serum metal ion concentrations such as; joint lubrication,^44^ activity level, ASA class, and primary diagnosis. Not taking these variables into account reduces the strength and generalisability of our study together with the fact that we have only analysed 1 brand of MoM, i.e the BHR implant. It has been shown that wear is implant-model specific.^45^

As 10 patients with re-operated implants and no previous metal ion sampling were excluded, some patients with high concentrations were potentially missed. We also excluded 89 patients who received a contralateral MoM-HR before their metal ions were measured. However, we do not have any reason to believe those patients differed from the studied population.

The patient population operated on at our institution with a BHR had either been referred to our institution or had directly contacted us with an outspoken interest in being operated on with that specific implant. The population is, therefore, not representative of the whole population of THA-operated patients. However, since the main outcome of this study was metal ion levels in relation to cup positioning such bias does not seem to be important.

In concordance with previous reports, women had smaller component sizes and higher sCo and sCr than men.^13,17^ Smaller heads are subjected to a higher risk of edge-loading due to a shorter distance before the loaded surface engages the rim.^46^ In addition, the cup articular arc angle, the angle between the femoral component centre and the bearing surface on the acetabular component of BHR, also decreases with smaller head diameters reducing the distance before the loaded part of the head comes into contact with the cup edge.^34^ However, in our study femoral head size did not affect the OR of having sCo and/or sCr >5 µg/l (Table 3). Thus we believe there must be other factors associated with patient sex that could explain the higher OR in women. Hypothetically, a larger range of hip motion in women could be a contributing factor.^47^ Unfortunately, we did not measure the range of motion consistently in our follow-up. The impact of individual motion patterns has been previously noted.^48^

In conclusion, it seems possible to reduce the number of patients with high metal ion levels by careful patient selection and optimal implant positioning but there will still be patients at risk. Correct positioning of the BHR acetabular component seems to be of crucial importance in decreasing the risk of elevated serum metal ion levels, although a perfectly implanted BHR acetabular component in a presumably ideal patient does not eliminate the risk of excessive metal wear.
